# Assessment of mutagenic potential of puberulic acid contaminated in red yeast rice (beni-koji) health food supplements

**DOI:** 10.1093/mutage/geag005

**Published:** 2026-02-05

**Authors:** Kei-ichi Sugiyama, Ayako Furuhama, Katsuyoshi Horibata, Masataka Tsuda, Kazuki Izawa, Genichiro Tsuji, Yosuke Demizu, Kohei Matsushita, Takeshi Toyoda, Yoko Hirabayashi, Yoshiro Saito, Masamitsu Honma

**Affiliations:** Division of Genome Safety Science, Center for Biological Safety and Research, National Institute of Health Sciences, 3-25-26 Tonomachi, Kawasaki, Kanagawa 210-9501, Japan; Division of Genome Safety Science, Center for Biological Safety and Research, National Institute of Health Sciences, 3-25-26 Tonomachi, Kawasaki, Kanagawa 210-9501, Japan; Division of Genome Safety Science, Center for Biological Safety and Research, National Institute of Health Sciences, 3-25-26 Tonomachi, Kawasaki, Kanagawa 210-9501, Japan; Division of Genome Safety Science, Center for Biological Safety and Research, National Institute of Health Sciences, 3-25-26 Tonomachi, Kawasaki, Kanagawa 210-9501, Japan; Division of Genome Safety Science, Center for Biological Safety and Research, National Institute of Health Sciences, 3-25-26 Tonomachi, Kawasaki, Kanagawa 210-9501, Japan; Division of Organic Chemistry, National Institute of Health Sciences, 3-25-26 Tonomachi, Kawasaki, Kanagawa 210-9501, Japan; Division of Organic Chemistry, National Institute of Health Sciences, 3-25-26 Tonomachi, Kawasaki, Kanagawa 210-9501, Japan; Division of Pathology, Center for Biological Safety and Research, National Institute of Health Sciences, 3-25-26 Tonomachi, Kawasaki, Kanagawa 210-9501, Japan; Division of Pathology, Center for Biological Safety and Research, National Institute of Health Sciences, 3-25-26 Tonomachi, Kawasaki, Kanagawa 210-9501, Japan; Center for Biological Safety and Research, National Institute of Health Sciences, 3-25-26 Tonomachi, Kawasaki, Kanagawa 210-9501, Japan; Division of General Affairs, National Institute of Health Sciences, 3-25-26 Tonomachi, Kawasaki, Kanagawa 210-9501, Japan; Division of Genome Safety Science, Center for Biological Safety and Research, National Institute of Health Sciences, 3-25-26 Tonomachi, Kawasaki, Kanagawa 210-9501, Japan; Division of General Affairs, National Institute of Health Sciences, 3-25-26 Tonomachi, Kawasaki, Kanagawa 210-9501, Japan

**Keywords:** puberulic acid, red yeast rice, mutagenicity, Ames test, QSAR, TGR assay, *pig-a* assay

## Abstract

In March 2024, a food poisoning incident in Japan was traced to red yeast rice (beni-koji) supplements contaminated with puberulic acid (PA), a mycotoxin produced by *Penicillium adametzioides*. Although PA was implicated in renal dysfunction in both humans and rats, its mutagenic potential had not been clarified. Here, we comprehensively assessed the mutagenicity of PA using a tiered approach that combined *in silico*, *in vitro*, and *in vivo* methods. *In silico* quantitative structure–activity relationship analyses predicted PA to be mutagenic, and *in vitro* Ames tests confirmed the positive finding. In contrast, *in vivo* assays, including the transgenic rodent gene mutation assay in mice and the *Pig-a* assay in rats, demonstrated no induction of mutations in kidney, glandular stomach, and blood cells, even at high exposure levels. Collectively, these findings indicate that PA is mutagenic *in vitro* bacterial tests, but not in mammalian *in vivo* systems, suggesting that the overall concern for mutagenicity in humans is low.

## Introduction

In late March 2024, a food poisoning incident occurred in Japan, characterized primarily by renal dysfunction following the consumption of a dietary supplement containing red yeast rice (beni-koji) [1–5]. To date, ~2700 individuals have consulted physicians, and >550 patients have been hospitalized and treated [1]. The dietary supplement in question was immediately recalled, and subsequent analysis suggested that puberulic acid (PA), derived from the mold *Penicillium adametzioides*, was responsible for the renal toxicity. Because PA was detected only in certain lots of the supplements, it was hypothesized that *Penicillium* contamination occurred at a stage during the cultivation of the red yeast, resulting in the production of PA, which was then included in the supplement [6].

Puberulic acid, with the IUPAC name 4,5,6-trihydroxy-3-oxocyclohepta-1,4,6-triene-1-carboxylic acid, is a mycotoxin possessing a tropolone structure (Table 1). Although PA produced by *Penicillium* species has been reported to exhibit bactericidal activity against gram-positive bacteria and antimalarial activity against *Plasmodium parasites* [7, 8], little is known about its other biological activities or its toxicity in mammals, including humans. In the present incident, the reported health effects in humans were predominantly cases of renal impairment. At the request of the Ministry of Health, labor, and Welfare (MHLW), we conducted a 28-day subacute toxicity study in rats, and demonstrated that PA also induced renal toxicity as the primary adverse effect in rats [9].

**Table 1:** The chemical structure of puberulic acid and results of QSAR prediction for its Ames mutagenicity.

**Chemical name**	**Structure**	**CAS#**	**CASE Ultra 1.9.0.4**		**Derek Nexus 9.3.0**	
			**Result**	**Probability (%)**	**Results**	**Comments**
Puberulic acid (PA)		99-23-0	Positive	56.3	Inactive	No misclassified or unclassified features
	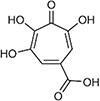					

Given that PA is a substance with previously unknown toxicological properties, however, it is necessary to consider other potential toxicities as well. More than 2700 individuals have already reported health complaints following ingestion of the implicated red yeast rice supplements, and the MHLW has received over 90 000 consultations regarding the consumption of the supplements [1]. This suggests that a substantial number of people were exposed to PA without experiencing noticeable symptoms. However, there is no assurance that these individuals will remain free of health problems in the future. It is therefore important to clarify the toxicological profile of PA and to assess potential long-term health risks.

Before administering a new chemical substance (a new drug candidate compound) to humans for the first time, it is essential to conduct nonclinical safety studies, including general toxicity, safety pharmacology, and mutagenicity assessments, to identify potential adverse effects in advance. Mutagenicity assessment, in particular, is extremely important for predicting future cancer risks and the possibility of genetic disorders [10]. However, despite the fact that many people have been exposed to PA in this food poisoning incident, there is no information available on its mutagenicity.

In this study, we assessed the mutagenic potential of PA. Mutagenicity testing for novel chemical substances is generally performed using the Ames test first. However, since PA was not available at the time of this incident, we predicted Ames mutagenicity using *in silico* analysis based on quantitative structure–activity relationship (QSAR). Subsequently, we succeeded in synthesizing PA in sufficient quantities and conducted the aforementioned 28-day subacute toxicity study. In parallel, we conducted the Ames test and *in vivo* mutagenicity tests using transgenic rodent gene mutation (TGR) assay and *Pig-a* assay to comprehensively evaluate the mutagenicity of PA. This case illustrates an effective strategy for assessing the mutagenic potential of an unknown chemical substance when it is implicated in human health damage.

## Materials and methods

### Test chemicals

Puberulic acid (4,5,6-trihydroxy-3-oxocyclohepta-1,4,6-triene-1-carboxylic acid; CAS no. 99–23-0) was synthesized in the Division of Organic Chemistry in NIHS (Table 1). The detailed synthesis method and analytical method were described in the previous paper [9]. Three lots were provided: Lot No. 20240520 (98.8% purity), Lot No. 20240904 (98.5% purity), and Lot No. 20240707 (98.5% purity). Lot No. 20240520 was used for the Ames test, and Lot No. 20240904 for the TGR assay. Lot No. 20240707 was used for the 28-day subacute toxicity study described above, and samples from this study were used for the *Pig-a* assay.

### Quantitative structure–activity relationship analyses

CASE Ultra version 1.9.2.1 with the GT1_BMUT module model version 1.9.2.0.1553.450 is statistical-based QSAR software developed by MultiCASE Inc., USA [11, 12]. It uses a statistical method to automatically extract alerts based on training data via machine learning technology. The prediction result is calculated as a probability based on the information on the mutagenicity warning structures in the training dataset, returning as ‘positive’ if the positive probability is 50%–100%, ‘inconclusive’ if it is 40%–50%, and ‘negative’ if it is 0%–40%. If the query compound is an exact match with a compound in the training dataset, known information such as ‘known positive’ or ‘known negative’ is also reported. ‘Out of domain’ means that the result is unpredictable.

DEREK Nexus 6.4.2 (Nexus: 2.8.0, DEREK KB 2024 1.0) is a rule-based expert SAR system developed by Lhasa Limited, UK [13, 14]. When a query compound matches a structural alert, DEREK Nexus offers the relevant inference level (certain, probable, plausible, equivocal, doubted, improbable, open, or inactive), which indicates the likelihood that compounds in a class will be active in an Ames test. ‘Inactive’ was normally defined as negative. However, if there are misclassified or unclassified features, ‘inactive’ results should be investigated on the basis of the target features.

### Ames test

The Ames test was conducted by a contract research organization (CRO) in accordance with the principles of GLP (Good Laboratory Practice) compliance, and with the Occupational Safety and Health Act test guidelines using a 20 min preincubation method [15]. This test guideline requires the use of five bacterial strains (*Salmonella typhimurium* TA100, TA98, TA1535, TA1537, and *Escherichia coli WP2 uvrA*) in both the presence and absence of S9-mix including 10% of rat S9, similar to the Organization for Economic Cooperation and Development (OECD) guideline TG471 [16]. The positive criterion was a two-fold increase in the number of revertant colonies over the control in at least one Ames test strain in the presence or absence of S9-mix. Dose-dependence and reproducibility were also considered in the final judgment.

### Transgenic rodent gene mutation assay using Muta™ mice

TGR assays were conducted by a CRO in accordance with the principles of GLP, and with the OECD Test Guideline TG488 [17]. Animals were treated in accordance with regulations of the Animal Care and Use Committees of the CRO and the NIHS, Japan.

Male and female Muta™ Mice (CD2-LacZ80/HazfBR) were purchased at 8 weeks of age from Japan SLC, Inc. (Sizuoka, Japan). In the dose-finding study, three male and female mice each were treated with PA at 3.00, 10.0, 30.0, or 100 mg/kg/day by oral gavage for 15 days, with 0.5 w/v% methyl cellulose used as a vehicle. In the TGR study, six female mice each were treated with PA at 1.25, 2.50, or 5.00 mg/kg/day for 28 days. Three days after the final treatment in the TGR study, the kidney and glandular stomach were each collected and stored. As a positive control group, mice were treated with *N-ethyl-N-nitrosourea* (ENU) at 100 mg/kg/day by intraperitoneal (*i.p.*) injection for two consecutive days. Genomic DNA was extracted from the kidney and glandular stomach (whole tissue) using the phenol/chloroform method. Transgenes were rescued via an *in vitro* packaging reaction using Transpack Packaging Extracts (Agilent Technologies, CA, USA). Mutant frequencies (MFs) were estimated via the *lacZ* positive selection method [18, 19]. Five mice each from the three dose groups were used for mutation assays. MFs were statistically analyzed using Dunnett’s test to compare treated groups against the vehicle control group and using Student’s or Aspin-Welch’s t-test to compare the positive control group against the vehicle control. A significance level of 5% was adopted with two-tailed tests.

### *Pig-a* assay

Blood samples for the *Pig-a* assay of PA were obtained from the previous a 28-day subacute toxicity study in rats [9]. In this study, male and female rats (five rats each dose group) were administered PA *via* oral gavage at doses of 1, 3, and 10 mg/kg/day for males, and 0.3, 1, and 3 mg/kg/day for females, for 28 consecutive days. On Day 29, the animals were anesthetized, and blood samples were collected from the abdominal aorta. Additionally, blood samples were collected from male and female animals in the high-dose recovery groups 14 days after the end of dosing (Day 43). Negative (PBS) and positive (ENU; 20 mg/kg/day) control samples from male rats were prepared separately.

*Pig-a* assay was conducted according to the OECD test guideline 470 and the recommendation by the International Workshop on Genotoxicity Testing (IWGT) Workgroup for the in vivo *Pig-a* assay [20, 21]. Briefly, 3 μL of blood was labeled with anti-rat CD59 (0.5 μg) and anti-rat erythroid marker (0.133 μg) antibodies. Approximately 1 × 10^6^ erythroid marker-positive cells were analyzed using a FACS Canto II flow cytometer (BD Biosciences) for the presence of surface CD59, and the *Pig-a* MFs were calculated [22]. The *Pig-a* MFs were analyzed by Bartlett’s test for homogeneity of variance among the groups. If the group variance was determined to be homogeneous, Dunnett’s multiple comparison test was employed. If Bartlett’s test indicated heterogeneous variance, Steel’s test was used. Significance was evaluated at the 5% level using a one-tailed test for increases relative to the vehicle control.

## Results

### QSAR analysis

We used two QSAR tools, CASE Ultra and DEREK Nexus, to predict the Ames mutagenicity of PA (Table 1). CASE Ultra calculated a positive probability of 56.3%, resulting in a positive result. DEREK Nexus, on the other hand, predicted as inactive, a negative result, with no misclassified or unclassified features. According to the DEREK Nexus output, this indicates that the query structure does not match any DEREK structure alerts or examples showing activity in the Ames test and the query structure contains no misclassified or unclassified or features.

### Ames test

Prior to the main study, a dose-finding test was conducted at 5000, 1250, 313, 78.1, 19.5, 4.88, and 1.22 μg/plate to determine the test dose in the main study. The number of revertant colonies increased in all strains, regardless of whether metabolic activation was present or absent. With the exception of *E. coli WP2 uvrA* without metabolic activation, a dose-dependent increase in the number of revertant colonies was observed, more than double the negative control value in all studies (Supplementary Data 1).

**Figure 1:**
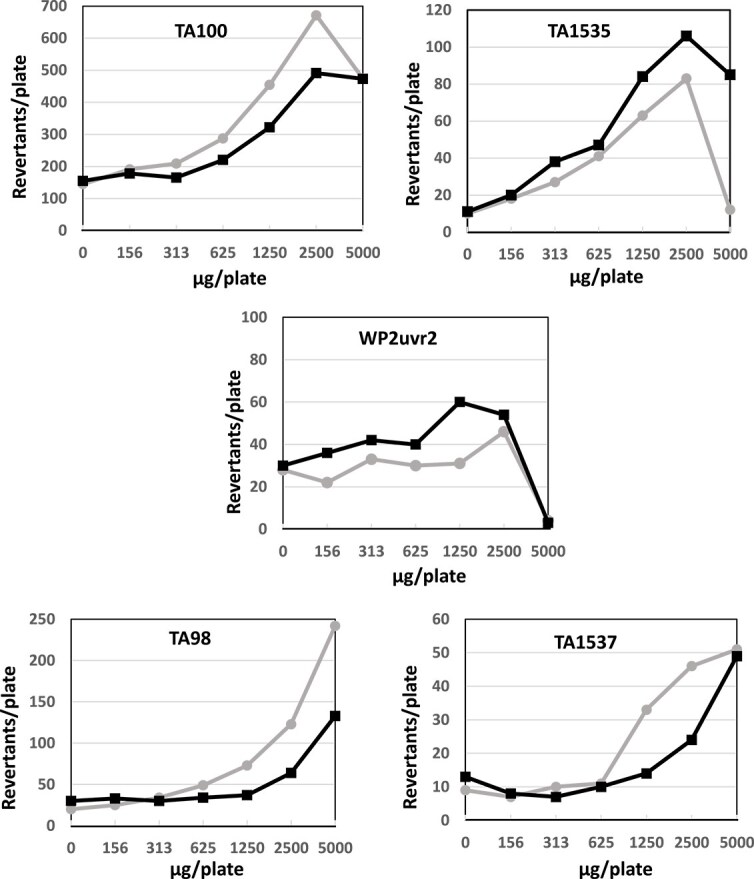
Mutagenicity of puberulic acid in the Ames test using five strains (TA100, TA1535, Wp2uvr2, TA98, TA1537) with or without S9-mix. Gray lines with circle means without S9-mix. Black line with square means with S9-mix.

The main study also yielded reproducible results. The number of revertant colonies increased in all strains with or without metabolic activation, and a dose-dependent increase in the number of revertant colonies, more than double the negative control value, was observed in all strains except *E. coli WP2 uvrA* without metabolic activation (Fig. 1 and Supplementary Data 1). The maximum relative activity value (MRAV), defined as the maximum number of induced revertant colonies per mg in all strains and treatments, was calculated to 278 (1250 μg/plate, TA100 without metabolic activation in dose-finding study).

### TGR assay

The dose-finding study was conducted using three male and female Muta™ Mice each at doses of 3.00, 10.0, 30.0, or 100 mg/kg. All animals in the 100 and 30 mg/kg groups died or became moribund until Day 8. In the 10.0 mg/kg group, significant weight loss, kidney edema, and discoloration were observed in both males and females. In the 3.00 mg/kg group, no changes in general condition or significant weight loss were observed in either sex. Based on these results, 5.00 mg/kg/day was considered to be near the maximum tolerated dose (MTD). Furthermore, because a previous 28-day subacute toxicity study in rats showed greater nephrotoxicity in females than in male [9], females were selected for the TGR study.

In the TGR study, no changes in general condition or weight progression were observed in any of the test substance groups. Necropsy revealed discoloration of the kidneys in one of six animals in the 5.00 mg/kg/day group. No notable changes were observed in other treatment groups. Since five mice were available for evaluation in all treatment groups, genomic DNA was extracted from the kidney and glandular stomach, and the MFs were evaluated at three doses: 1.25, 2.50, and 5.00 mg/kg/day. There was no significant increase in MFs in the kidneys and glandular stomach of all PA-treated mice compared with tissues from negative control animals (Tables 2 and 3). In contrast, a significant increase in MFs was observed in the kidneys and glandular stomach of ENU-treated mice from positive control group (*P* ≤ 0.05).

**Table 2:** Induction of mutation in kidney of Muta^TM^ Mouse treated with Puberulic acid.

**Substance**	**Dose (mg/kg/day, p.o.)**	**Animal ID No.**	**Number of plaque forming units**	**Number of mutant Plaques**	**Mutant Frequency (X10** ^**−6**^ **)**	**Mutant Frequency (X10** ^**−6**^ **) Average ± S.D.**
0.5% MC	0	3001	7,84,800	15	19.1	30.0 ± 7.5
		3002	10,80,900	35	32.4	
		3003	11,99,700	46	38.3	
		3004	9,94,500	34	34.2	
		3005	9,54,900	25	26.2	
Puberulic acid	1.25	3201	11,62,800	24	20.6	35.0 ± 11.8
		3202	14,93,100	49	32.8	
		3203	11,47,500	46	40.1	
		3204	20,02,500	59	29.5	
		3205	15,40,800	80	51.9	
	2.5	3301	17,46,000	67	38.4	48.8 ± 37.0
		3302	13,69,800	47	34.3	
		3303	12,92,400	148	114.5	
		3304	6,81,300	18	26.4	
		3305	11,13,300	34	30.5	
	5	3401	9,06,300	24	26.5	38.1 ±10.1
		3403	11,87,100	40	33.7	
		3404	10,76,400	57	53	
		3405	9,63,000	41	42.6	
		3406	13,45,500	47	34.9	
ENU	100	3501	11,00,700	136	123.6	127.1 ± 41.3^*^
		3502	13,59,000	267	196.5	
		3503	14,26,500	159	111.5	
		3504	11,11,500	131	117.9	
		3505	7,30,800	63	86.2	

0.5% MC: Negative control (0.5 w/v% Methylcellulose solution, 10 mL/kg)

ENU: Positive control (N -ethyl-N -nitrosourea, 10 mL/kg, i.p., dose once a day, for 2 days, expression period; 10 days)

^*^: Significant difference from negative control (p<0.05)

**Table 3:** Induction of mutation in glandular stomach of Muta^TM^ Mouse treated with Puberulic acid.

**Substance**	**Dose (mg/kg/day, p.o.)**	**Animal ID No.**	**Number of plaque forming units**	**Number of mutant Plaques**	**Mutant Frequency (X10** ^**−6**^ **)**	**Mutant Frequency (X10** ^**−6**^ **) Average ± S.D.**
0.5% MC	0	3001	3,30,300	7	21.2	26.8 ± 5.3
		3002	5,91,300	18	30.4	
		3003	8,07,300	18	22.3	
		3004	6,07,500	16	26.3	
		3005	8,56,800	29	33.8	
			
Puberulic acid	1.25	3201	7,28,100	24	33	32.7 ± 7.6
		3202	11,25,000	44	39.1	
		3203	6,55,200	16	24.4	
		3204	11,24,100	29	25.8	
		3205	12,80,700	53	41.4	
			
	2.5	3301	12,97,800	47	36.2	32.5 ± 7.0
		3302	6,34,500	15	23.6	
		3303	7,10,100	19	26.8	
		3304	5,44,500	22	40.4	
		3305	7,06,500	25	35.4	
	5	3401	17,43,300	52	29.8	35.5 ±14.6
		3403	18,04,500	103	57.1	
		3404	8,56,800	32	37.3	
		3405	6,57,900	11	16.7	
		3406	8,99,100	33	36.7	
ENU	100	3501	10,57,500	463	437.8	472.3 ± 71.8^*^
		3502	12,62,700	694	549.6	
		3503	12,84,300	662	515.5	
		3504	3,53,700	174	491.9	
		3505	6,08,400	223	366.5	

0.5% MC: Negative control (0.5 w/v% Methylcellulose solution, 10 mL/kg)

ENU: Positive control (N -ethyl-N -nitrosourea, 10 mL/kg, i.p., dose once a day, for 2 days, expression period; 10 days)

^*^: Significant difference from negative control (p<0.05)

### Pig-a assay

**Figure 2:**
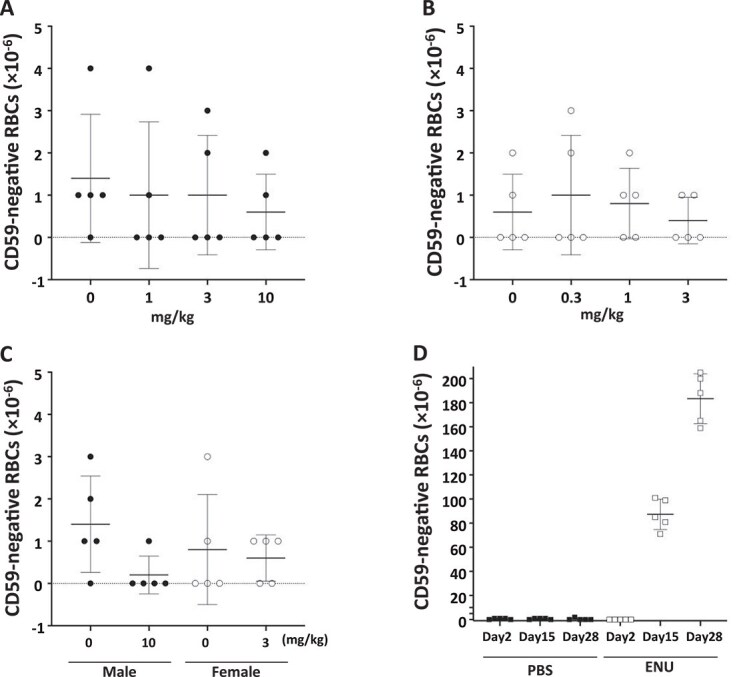
The results of *pig-a* assays in males on day 29 (A), *pig-a* assays in females on day 29 (B), *pig-a* assays in males and females on day 43 (C). The vertical axis represents the frequency of CD59-deficient cells (*pig-a* MFs), and the horizontal axis represents the dose of puberulic acid administered. The individual data are expressed as dot plots (dots (●) for males and dots (◯) for females, respectively), along with the mean ± SD. *Pig-a* MFs of male rats treated with ENU; 20 mg/kg/day (positive control) and with PBS (negative control) are shown as dot plots (dots (□) for ENU and dots (■) for PBS, respectively), along with the mean ± SD (D).

*A Pig-a* assay for PA was conducted using blood samples obtained from a 28-day subacute toxicity study in rats [9]. No animals died in any treatment group, and blood samples were taken one day after the final dose (Day 29) and after recovery (Day 43). The *Pig-a* assay did not show any increase in the PA treatment groups compared to the negative control group (Fig. 2A–C). The positive control ENU treatment group showed a strong positive result (Dunnett’s multiple comparison test; *P* ≤ 0.05), so the study was deemed to have been conducted appropriately (Fig. 2D).

## Discussion

The dietary supplement in question is a supplement (functional food) containing red rice koji polyketides, which inhibit cholesterol synthesis in the body and lower LDL cholesterol [23]. The certain lots caused health problems, primarily kidney dysfunction, was found to contain impurities: two unknown compounds with a basic structure similar to that of monacolin K, the active ingredient in the supplement [24], as well as puberulic acid (PA). PA is a mycotoxin produced by the mold *Penicillium adametzioides* that was contaminated during the manufacturing process of the beni-koji supplement [6].

The manufacturer reported that PA was suspected to be the cause of the kidney dysfunction, but PA had been a virtually unknown chemical substance and was unavailable as a substance at the time of the incident. Therefore, it took >6 months to successfully chemically synthesize sufficient quantities of PA and conduct toxicity studies. In a 28-day subacute toxicity study using rats, this synthesized PA caused obvious kidney damage and gastric mucosal damage, but no other significant toxicity was observed [9]. However, this does not mean that the toxic effects of PA have been fully elucidated. In this study, we comprehensively assessed the mutagenic potential of PA. If PA is suspected to be mutagenic in human, an appropriate carcinogenic risk assessment may be required.

For the comprehensive mutagenicity assessment of PA, we followed the ICH-M7(R2) guideline, which is ‘Assessment and Control of DNA Reactive (Mutagenic) Impurities in Pharmaceuticals to Limit Potential Carcinogenic Risk’ [25]. This guideline recommends a tiered approach to assessing the mutagenicity of pharmaceutical impurities. It includes: (1) predicting Ames mutagenicity using the *in silico* method QSAR; (2) evaluating *in vitro* mutagenicity using the Ames test; and (3) evaluating *in vivo* gene mutagenicity using animals.

QSAR is useful in cases where actual biological testing is difficult, such as with pharmaceutical impurities. In this case, PA was initially unavailable, forcing us to first conduct QSAR prediction using two QSAR tools: a statistical based QSAR tool (CASE Ultra) and a rule-based expert QSAR tool (DEREK Nexus). The result was positive in CASE Ultra and negative in DEREK Nexus (Table 1). The ICH-M7(R2) guideline requires negative results in both QSARs to rule out mutagenicity. These results suggest that PA may possess Ames mutagenicity. Furthermore, while no information exists on the Ames mutagenicity of PA, tropolone tosylate (CAS no. 38768–08-0), which shares the same basic troponin structure as PA, is registered in our Ames test database as an Ames-positive substance [26, 27]. These *in silico* analyses suggest that PA may have Ames mutagenicity.

Subsequently, we successfully synthesized PA and performed the Ames test. All four *Salmonella* strains showed revertants at rates more than twice those of the negative control, with clear dose-dependent responses (Fig. 1), confirming the *in silico* predictions. Based on these results, we concluded that PA was positive in the Ames test. Furthermore, The MRAV was calculated to be 278 (1250 μg/plate, TA100 without metabolic activation in a dose-finding experiment), indicating moderate mutagenic activity. Note that a specific activity greater than 1000 is defined as a strong mutagen under Industrial Safety and Health Law in Japan [15].

Following the positive *in vitro* Ames test results, we performed the *in vivo* TGR test. Because the 28-day repeated subacute toxicity test showed obvious renal damage and gastric mucosal damage, we examined gene mutation induction in the kidney and glandular stomach. However, no mutation induction was observed in both tissues or at any tested dose (Tables 2 and 3). We also performed the *Pig-a* assay using blood samples from the 28-day subacute toxicity study (Fig. 2), but no induction of mutant cells was observed. Furthermore, we performed the error-corrected next generation sequencing (ecNGS) analysis on kidney tissue from two males treated with the highest dose (10 mg/kg bw/day) and two control rats in the 28-day subacute toxicity test to compare the MFs [28]. A marked increase in mutations was not observed (Supplementary Data 2). These findings suggested that PA did not exhibit mutagenicity in the kidney, glandular stomach, and blood cells under these administration conditions, and is not mutagenic *in vivo*.

Based on these results, we concluded that PA is mutagenic *in vitro* bacterial tests, but not *in vivo* animal studies, suggesting low carcinogenic concern in humans. This is not surprising, given that 40%–50% of chemical substances that positive in the Ames test were judged to be negative in *in vivo* genotoxicity tests [29], and that 35%–40% Ames positive chemicals were judged as negative in rodent carcinogenicity tests [30]. *In silico* (QSAR) and *in vitro* (Ames) tests are screening tests that detect a wide range of potentially mutagenic substances and then confirm the results in *in vivo* tests. This tiered approach strategy is rational and scientific from the perspectives of both ensuring safety and efficiency.

On the other hand, this strategy was based on the mutagenicity assessment approach used in ICH-M7(R2), which was a guideline for the assessment and control of small amounts of mutagenic Impurities in pharmaceuticals, and its scope of application is generally limited to daily exposures of 1 mg/kg or less [25]. In this food poisoning incident, however, it is probable that many of the individuals who reported kidney dysfunction had consumed more than this amount. For impurities with high daily exposure levels and new pharmaceuticals, assessment of not only mutagenicity but also other genotoxicity such as clastogenicity is required. In some cases, it may be warranted to conduct *in vitro* chromosomal aberration tests or *in vivo* micronucleus assays to further verify safety [31].

## Conclusion

We conducted a comprehensive assessment of the mutagenicity of PA, which was responsible for serious health effects following the consumption of beni-koji supplements. PA was positive in both *in silico* QSAR analyses and the *in vitro* Ames test, but consistently negative in *in vivo* TGR assay in the kidney and glandular stomach, as well as in *in vivo Pig-a assay* in blood cells. These findings indicate that PA is not mutagenic to mammals and poses a low carcinogenic risk to humans. We propose that this comprehensive, tiered mutagenicity assessment strategy—combining *in silico*, *in vitro*, and *in vivo* approaches—represents an ideal framework for clarifying the actual health effects caused by chemicals and for providing robust information on mutagenicity in health risk assessments.

## Supplementary Material

Supplementay_data_1_1001_geag005

Supplementary_data_2_1001_geag005

## Data Availability

The data can be made available from the corresponding author on reasonable request.
