# Supplementary material for: Assessment of mutagenic potential of puberulic acid contaminated in red yeast rice (beni-koji) health food supplements
Source: Mutagenesis. 2026 Feb 5;41(3):110–8. doi: 10.1093/mutage/geag005 (PMC13107177; doi:10.1093/mutage/geag005)
Supplement: Supplementay_data_1_1001_geag005 [file supplementay_data_1_1001_geag005.pdf]

Supplementary data 1

Dose-Finding Test

Metabolic activation	Dose (µg/plate)	Colonies/plate									
		Base-substitution						Frameshift			
		TA100		TA1535		WP2uvrA		TA98		TA1537	
S9 mix (-)	Negative control (DMSO)	118		7		30		19		10	
		117	(118)	7	(7)	28	(29)	22	(21)	7	(9)
	1.22	130		10		28		22		8	
		110	(120)	3	(7)	32	(30)	19	(21)	5	(7)
	4.88	102		9		36		22		5	
		105	(104)	8	(9)	34	(35)	24	(23)	13	(9)
	19.5	106		11		32		15		6	
		127	(117)	7	(9)	31	(32)	18	(17)	6	(6)
	78.1	128		15		27		36		15	
		119	(124)	10	(13)	30	(29)	35	(36)	8	(12)
	313	196		28		32		52		13	
		179	(188)	22	(25)	27	(30)	26	(39)	17	(15)
	1250	453		60*		50		63		35*	
		479	(466)	49*	(55)	48	(49)	79	(71)	26*	(31)
	5000	462*		10*		5*		202*		50*	
		549*	(506)	21*	(18)	2*	(4)	233*	(218)	41*	(46)
S9 mix (+)	Negative control (DMSO)	122		9		23		26		13	
		129	(126)	14	(12)	27	(25)	31	(29)	6	(10)
	1.22	150		7		25		24		10	
		142	(146)	5	(6)	28	(27)	32	(28)	13	(12)
	4.88	152		5		39		24		7	
		129	(141)	5	(5)	20	(30)	34	(29)	5	(6)
	19.5	134		10		27		20		5	
		149	(142)	13	(12)	26	(27)	35	(28)	6	(6)
	78.1	153		14		33		35		14	
		163	(158)	11	(13)	24	(29)	33	(34)	9	(12)
	313	187		19		27		28		13	
		175	(181)	16	(18)	33	(30)	33	(31)	9	(11)
	1250	294		56		43		30		11	
		329	(312)	78	(67)	58	(51)	30	(30)	8	(10)
	5000	548*		27*		8*		121*		55*	
		453*	(501)	19*	(23)	7*	(8)	109*	(115)	42*	(49)
S9 mix (-)	Positive controls	AF-2		SAZ		AF-2		AF-2		ICR-191	
	Dose (µg/plate)	0.01		0.5		0.01		0.1		1.0	
	Colonies/plate	710		211		101		479		1417	
		693	(702)	206	(209)	133	(117)	500	(490)	1444	(1431)
S9 mix (+)	Positive controls	B[a]P		2AA		2AA		B[a]P		B[a]P	
	Dose (µg/plate)	5.0		2.0		10.0		5.0		5.0	
	Colonies/plate	1171		245		710		293		96	
		1231	(1201)	233	(239)	667	(689)	290	(292)	67	(82)

AF-2: 2-(2-Furyl)-3-(5-nitro-2-furyl)acrylamide
SAZ: Sodium azide
ICR-191:6-Chloro-9-[3-(2-chloroethylamino)propylamino]-2-methoxyacridine dihydrochloride
B[a]P: Benzo[a]pyrene
2AA: 2-Aminoanthacene
(): average value
*: Growth inhibition

Main Test

Metabolic activation	Dose (µg/plate)	Colonies/plate									
		Base-substitution						Frameshift			
		TA100		TA1535		WP2uvrA		TA98		TA1537	
	Negative control (DMSO)	139		9		27		16		10	
		152	(146)	10	(10)	29	(28)	24	(20)	8	(9)
	39.1	NT		10		NT		NT		14	
				9	(10)					10	(12)
	78.1	NT		17		NT		NT		5	
				14	(16)					10	(8)

S9 mix (-)	156	180<br>202	(191)	13<br>22	(18)	21<br>23	(22)	23<br>27	(25)	6<br>6	(6)
	313	202<br>216	(209)	34<br>20	(27)	39<br>27	(33)	28<br>40	(34)	9<br>11	(10)
	625	269<br>305	(287)	39<br>42	(41)	27<br>32	(30)	43<br>54	(49)	8<br>13	(11)
	1250	456<br>452	(454)	59*<br>66*	(63)	34<br>28	(31)	78<br>67	(73)	34*<br>32*	(33)
	2500	643<br>698	(671)	75*<br>91*	(83)	54<br>37	(46)	120<br>126	(123)	51*<br>41*	(46)
	5000	370*<br>578*	(474)	19*<br>6*	(12)	6*<br>1*	(4)	245*<br>238*	(242)	50*<br>51*	(51)
S9 mix (+)	Negative control (DMSO)	155<br>147	(155)	9<br>12	(11)	31<br>29	(30)	28<br>31	(30)	12*<br>13*	(13)
	156	176<br>179	(178)	18<br>22	(20)	35<br>36	(36)	33<br>32	(33)	8<br>8	(8)
	313	169<br>160	(165)	34<br>42	(38)	42<br>42	(42)	28<br>32	(30)	4<br>9	(7)
	625	216<br>223	(220)	43<br>51	(47)	42<br>38	(40)	25<br>42	(34)	11<br>8	(10)
	1250	299<br>344	(322)	76<br>91	(84)	53<br>67	(60)	31<br>43	(37)	11<br>17	(14)
	2500	457<br>524	(491)	96<br>116	(106)	53<br>54	(54)	63<br>64	(64)	22<br>25	(24)
	5000	447*<br>498*	(473)	101*<br>68*	(85)	5*<br>0*	(3)	119*<br>146*	(133)	54*<br>44*	(49)
S9 mix (-)	Positive controls	AF-2		SAZ		AF-2		AF-2		ICR-191	
	Dose (µg/plate)	0.01		0.5		0.01		0.1		1.0	
	Colonies/plate	576<br>557	(567)	267<br>295	(281)	86<br>98	(92)	461<br>461	(461)	1506<br>1628	(1567)
S9 mix (+)	Positive controls	B[a]P		2AA		2AA		B[a]P		B[a]P	
	Dose (µg/plate)	5.0		2.0		10.0		5.0		5.0	
	Colonies/plate	1048<br>1245	(1147)	222<br>207	(215)	736<br>779	(758)	300<br>300	(300)	78<br>84	(81)

AF-2: 2-(2-Furyl)-3-(5-nitro-2-furyl)acrylamide
SAZ: Sodium azide
ICR-191:6-Chloro-9-[3-(2-chloroethylamino)propylamino]-2-methoxyacridine dihydrochloride
B[a]P: Benzo[a]pyrene
2AA: 2-Aminoanthacene
(): average value
*: Growth inhibition
NT: Not tested
