# Supplementary material for: Assessment of mutagenic potential of puberulic acid contaminated in red yeast rice (beni-koji) health food supplements
Source: Mutagenesis. 2026 Feb 5;41(3):110–8. doi: 10.1093/mutage/geag005 (PMC13107177; doi:10.1093/mutage/geag005)
Supplement: Supplementary_data_2_1001_geag005 [file supplementary_data_2_1001_geag005.docx]

**Supplementary data 2**

The ecNGC analysis was performed using kidney tissue from two male rats (PA132, PA133) administered the highest dose (10 mg/kg bw/day) and two control rats (PA101, PA102) in a 28-day subacute toxicity study. Genomic DNA was extracted from the tissue samples using the DNeasy® Blood & Tissue kit (QIAGEN). Genomic DNA was fragmented using NEBNext® dsDNA Fragmentase® (NEB), and sequencing libraries were generated using TruSeq DNA PCR-Free (Illumina). Sequencing was performed using Illumina NovaSeq. The reference genome sequence used for data analysis was the Rattus norvegicus genome assembly GRCr8 (GCA_036323735.1), and mutation detection was performed using PECC-Seq. Statistical tests were performed using t-tests.

**Table S2 Mutation frequencies (MFs)**

| Samples | MFs |
| --- | --- |
| PA101（control） | 0.93×10^-7^ |
| PA102（control） | 0.95×10^-7^ |
| PA132（10 mg/kg bw/day | 0.92×10^-7^ |
| PA133（10 mg/kg bw/day） | 1.23×10^-7^ |

|  |  |  |
| --- | --- | --- |
|  |  |  |

**Fig S2 The types of various mutation obtained from each sample and their MFs**
